# Clinical investigation: thyroid function test abnormalities in cardiac arrest associated with acute coronary syndrome

**DOI:** 10.1186/cc3727

**Published:** 2005-06-09

**Authors:** Kenan Iltumur, Gonul Olmez, Zuhal Arıturk, Tuncay Taskesen, Nizamettin Toprak

**Affiliations:** 1 Assistant Professor, Dicle University Medical Faculty Department of Cardiology, Diyarbakir, Turkey; 2 Assistant Professor, Dicle University Medical Faculty Department of Anesthesia and Reanimation, Diyarbakir, Turkey; 3 Resident, Dicle University Medical Faculty Department of Cardiology, Diyarbakir, Turkey; 4 Professor, Dicle University Medical Faculty Department of Cardiology, Diyarbakir, Turkey

## Abstract

**Introduction:**

It is known that thyroid homeostasis is altered during the acute phase of cardiac arrest. However, it is not clear under what conditions, how and for how long these alterations occur. In the present study we examined thyroid function tests (TFTs) in the acute phase of cardiac arrest caused by acute coronary syndrome (ACS) and at the end of the first 2 months after the event.

**Method:**

Fifty patients with cardiac arrest induced by ACS and 31 patients with acute myocardial infarction (AMI) who did not require cardioversion or cardiopulmonary resuscitation were enrolled in the study, as were 40 healthy volunteers. The patients were divided into three groups based on duration of cardiac arrest (<5 min, 5–10 min and >10 min). Blood samples were collected for thyroid-stimulating hormone (TSH), tri-iodothyronine (T_3_), free T_3_, thyroxine (T_4_), free T_4_, troponin-I and creatine kinase-MB measurements. The blood samples for TFTs were taken at 72 hours and at 2 months after the acute event in the cardiac arrest and AMI groups, but only once in the control group.

**Results:**

The T_3 _and free T_3 _levels at 72 hours in the cardiac arrest group were significantly lower than in both the AMI and control groups (*P *< 0.0001). On the other hand, there were no significant differences between T_4_, free T_4 _and TSH levels between the three groups (*P *> 0.05). At the 2-month evaluation, a dramatic improvement was observed in T_3 _and free T_3 _levels in the cardiac arrest group (*P *< 0.0001). In those patients whose cardiac arrest duration was in excess of 10 min, levels of T_3_, free T_3_, T_4 _and TSH were significantly lower than those in patients whose cardiac arrest duration was under 5 min (*P *< 0.001, *P *< 0.001, *P *< 0.005 and *P *< 0.05, respectively).

**Conclusion:**

TFTs are significantly altered in cardiac arrest induced by ACS. Changes in TFTs are even more pronounced in patients with longer periods of resuscitation. The changes in the surviving patients were characterized by euthyroid sick syndrome, and this improved by 2 months in those patients who did not progress into a vegetative state.

## Introduction

The most common reason for cardiac arrest in adults is coronary heart disease [1]. In particular, sudden and unexpected cardiac arrest may occur after an acute myocardial infarction (AMI) [2,3]. Prompt intervention (such as cardioversion and cardiopulmonary resuscitation [CPR]) can successfully resuscitate cardiac arrest patients [4,5]. Cardiac output rarely reaches 25% of its normal level during CPR in cardiac arrest, which renders cerebral blood flow inadequate. Cerebral blood flow is less than 30% at this stage [6], which results in varying degrees of hypoxic encephalopathy [7].

The hypophysis and hypothalamus are intracerebral organs, and if blood flow is inadequate then the function of these organs may be critically impaired. It is known that the hypothalamus-pituitary-thyroid axis is affected in patients with brain death. Although the underlying mechanism has not been elucidated, it is generally considered an endocrine abnormality characterized by 'euthyroid sick syndrome' (ESS) [8]. It is also known that certain nonthyroid critical conditions, including heart disease, may also lead to ESS [9-19]. The ESS (or the 'low T_3 _syndrome') occurs as a result of impairment in normal feedback response due to low tri-iodothyronine (T_3_) levels and disruption in conversion of the precursor hormone thyroxine (T_4_) to T_3_. Furthermore, the inactive metabolite reverse T_3 _accumulates in ESS [13,19].

Thyroid hormones have a major impact on the cardiovascular system [20-22]. Low T_3 _concentrations are known to be major independent indicators of mortality in patients hospitalized for cardiac causes [23]. Previous studies [24-27] reported critical impairments in thyroid homeostasis during the acute stage of cardiac arrest. However, it is not certain how, for how long and in which patient population this critical condition occurs. In addition, to our knowledge, thyroid functions have not yet been systematically assessed in patients with cardiac arrest caused by acute coronary syndrome (ACS). In the present study, conducted in patients who were resuscitated following cardiac arrest caused by ACS, we evaluated alterations that occur in thyroid hormone metabolism during the acute stage of cardiac arrest and at the end of the first 2 months after the event.

## Materials and methods

A total of 50 patients with cardiac arrest caused by ACS (35 males and 15 females) who had been resuscitated (by cardioversion or CPR) and hospitalized in the intensive care unit (ICU) within the first 72 hours, and 31 AMI patients who did not require cardioversion or CPR (25 males and 6 females) were enrolled in the study, as were 40 healthy volunteers (28 males and 12 females). All patients or, in the case of unconsciousness, their closest relative signed a written informed consent form. The protocol was approved by the local ethics committee.

Patients were excluded if they were known to have thyroid function test (TFT) abnormalities that could not be related to AMI or cardiac arrest. We also excluded those patients who had previously suffered acute coronary events, who had previously undergone percutaneous transluminal coronary angioplasty or bypass surgery, who had a history of heart failure, and who received medication that could alter thyroid function, such as amiodarone and phenytoin (excluding β-blockers, heparin and dopamine), or who had comorbid conditions (malignancy, hepatic, or renal failure).

### Cardiac arrest group

Of the 50 patients (35 males and 15 females; mean age 59 ± 8 years) in the cardiac arrest group, 28 patients were resuscitated using CPR, whereas the remaining 22 patients only underwent cardioversion. In cardiac arrest patients, three subgroups were defined based on the duration of intervention in order to investigate whether this had any impact on TFTs: cardiac arrest group 1, <5 min (*n *= 24; mostly consisting of patients who underwent cardioversion); cardiac arrest group 2, 5–10 min (*n *= 14); and cardiac arrest group 3, >10 min (*n *= 12). Postischaemic anoxic encephalopathy (cerebral postresuscitation syndrome or disease) grading was done according to the classification reported by Maiese and Caronna [7]. The possible outcomes they distinguished are as follows: dead, decerebrate, persistent vegetative state, severe focal neurological deficit, amnesic syndrome and neurologically intact (but often with psychological changes).

Patients with cardiac arrest were followed up in the ICU until their cardiac function became stable. The patients received standard therapies, depending on the aetiology of cardiac arrest (ACS with or without ST-segment elevation). A total of 23 patients did not receive thrombolytic thera and the remaining 27 patients underwent thrombolytic therapy with streptokinase. The patients with severe arrhythmia were administered lidocaine, an antiarrhythmic agent. Furthermore, four patients received dopamine because of low blood pressure. All patients received therapy required to achieve a normal metabolic condition and acid–base balance.

### Acute myocardial infarction group

The AMI group included 31 (25 males and 6 females; mean age 57 ± 9 years) consecutive AMI patients admitted to the ICU within the first 12 hours after the event and who did not require cardioversion or CPR. Myocardial infarction was defined using the European Society of Cardiology/American College of Cardiology guidelines [28]. All patients received standard medical therapy, consisting of aspirin, heparin, intravenous nitrates and β-blockers, where it was not contraindicated. Furthermore, all patients with AMI were treated with streptokinase (1.5 million IU in 60 min). Continuous electrocardiogram telemetry monitoring was done in all patients during their stay in the coronary care unit.

### Control group

The control group included 40 volunteers (28 males and 12 females; mean age 58 ± 6 years) without angina pectoris and with the same age distribution and similar male/female ratios as the cardiac arrest and AMI groups. History, physical examination, electrocardiography, chest radiography and routine chemical analysis identified no evidence of coronary heart disease in these individuals.

### Laboratory measurements

Fasting blood samples were collected for thyroid hormone profile from cardiac arrest and AMI groups after an average period of 72 hours following the initial event. Blood samples were also taken during the first 12 hours in the AMI group. Furthermore, blood samples were collected again for follow-up assessment from surviving patients in both groups at the end of the second month. Fasting blood samples from the control individuals were collected once. Blood samples drawn from brachial vein were centrifuged, and measurements of T_3_, free T_3_, T_4_, free T_4 _and thyroid-stimulating hormone (TSH) were taken. Serum T_3_, free T_3_, T_4_, free T_4 _and TSH serum levels were assessed using a Roshe-170E modular analytics device (Roshe Diagnostics GmbH, Mannheim for USA, US Distributor: Roshe Diagnostics, Indianapolis, IN), employing the electrochemiluminescence method. The reference intervals for our laboratory are as follows: T_3_, 0.85–2.02 ng/ml; T_4_, 5.13–14.06 μg/dl; free T_3_, 0.18–0.46 ng/ml; free T_4_, 0.93–1.71 ng/dl; and TSH, 0.27–4.2 μIU/ml. Standard procedures were used to determine serum levels of creatine kinase-MB and troponin I.

Echocardiographic examination was performed with a HP SONOS 4500 (Agilent Technologies Andover, Canada), using a 3.5 or 2.5 MHz transducer. Echocardiographical images were obtained from parasternal and apical views. Parasternal long axis, short axis, and apical four chamber views were assessed according to the criteria recommended by the American Echocardiography Society (29). The left ventricular ejection fraction (LVEF) was assessed echocardiographically, using the Simpson biplane formula [29].

Patients remained in the ICU until they were stable in terms of their ischaemic heart disease. Those with complications other than ischaemic heart disease (severe neurological deficit, or persistent vegetative or decerebrate state) were monitored in neurology departments. Coronary angiography was performed if indicated in those patients whose condition became stable. Iopromid (Ultravist; 370 mg iodine/ml Schering Alman, Istanbul Turkey)) was used as the contrast medium in coronary angiography.

### Statistics

All values were expressed as mean ± standard deviation. The data were analyzed by analysis of variance for repeated measurements, followed by *post hoc *analysis for pairwise comparisons, and were corrected by Tukey test or paired t-test when indicated. *P *< 0.05 was considered statistically significant.

## Results

Although patients in the cardiac arrest group were older than the AMI patients and control individuals, the difference was not statistically significant (*P *> 0.05). Most of the patients were men. The patients in cardiac arrest group were classified according to the Maiese and Caronna classification as follows: 21 were neurologically intact, 13 were amnesic, four had severe neurological deficit, two were in a persistent vegetative state, eight were decerebrate and two were dead. Of the cardiac arrest patients, 23 had anterior myocardial infarction, nine had inferior myocardial infarction, 14 had inferior myocardial infarction with right ventricular involvement, and four had non-Q-wave myocardial infarction. The AMI group included 14 patients with anterior myocardial infarction, 10 with inferior myocardial infarction, and seven with inferior myocardial infarction with right ventricular involvement.

Of the cardiac arrest patients, the duration of intervention was under 5 min for 24 patients (22 underwent cardioversion), 5–10 min for 14 patients, and longer than 10 min for 12 patients. Although 22 of the cardiac arrest patients died within the first 2 months, only one patient died in the AMI group. Of the cardiac arrest patients who died, 11 had an intervention lasting longer than 10 min, eight had an intervention lasting 5–10 min, and three had an intervention lasting less than 5 min. It was observed that, although troponin and CK-MB levels were higher, LVEF was lower in the cardiac arrest group compared with those parameters for the AMI group (*P *< 0.0001, *P *< 0.05 and *P *< 0.05, respectively). The characteristics of the patients and control inidividuals are summarized in Table 1.

Coronary angiography was performed in a total of 37 patients. Of these patients, 15 were in the cardiac arrest group and 22 were in the AMI group. The mean volume of contrast medium used in coronary angiography was 110 ± 19 ml. In the statistical analysis applied, at the end of the second month the TFT results for patients undergoing angiography were similar to those in patients not undergoing angiography (angiography versus no angiography: T_3_, 1.16 ± 0.25 versus 1.12 ± 0.22 ng/ml; free T_3_, 0.29 ± 0.06 versus 0.28 ± 0.09 ng/ml; T_4_, 8.45 ± 2 versus 7.84 ± 1.99 μg/dl; free T_4_, 1.31 ± 0.19 versus 1.29 ± 0.26 ng/dl; TSH, 1.35 ± 0.73 versus 1.19 ± 0.61 μIU/ml; *P *> 0.05 for all comparisons).

**Figure 1:**
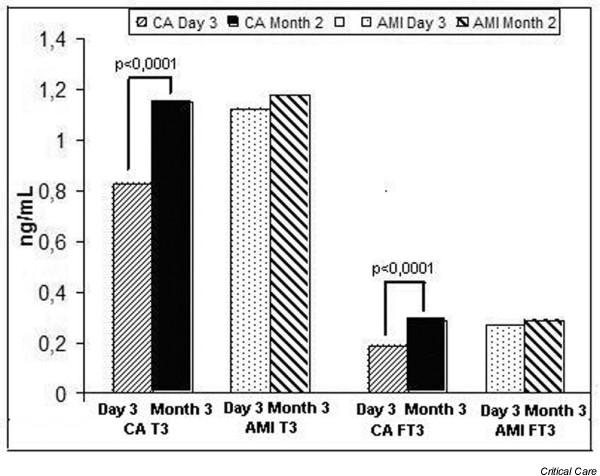
T_3 _and FT_3 _levels in the CA group had increased by the end of month 2.

The T_3 _and free T_3 _levels on day 3 in the cardiac arrest group were significantly lower than those in the AMI group and control group (*P *< 0.0001). In contrast, T_4_, free T_4 _and TSH levels did not differ significantly between groups (*P *> 0.05; Table 2). The cardiac arrest group had lower T_3 _(0.9 ± 0.31 versus 1.13 ± 0.24 ng/ml) and free T_3 _(0.22 ± 0.12 versus 0.29 ± 0.07 ng/ml) levels than did the AMI group on day 3, even when subgroups were analyzed and only the surviving patients were considered (for both, *P *< 0.01). However, at the 2-month follow-up visits, T_3 _and free T_3 _levels were found to have improved dramatically in the cardiac arrest group (*P *< 0.0001; Fig. 1 and Table 3).

When the subgroup of patients who underwent cardioversion alone was compared with the subgroup of patients who underwent CPR alone, it was observed that T_3 _and free T_3 _levels were lower in the CPR subgroup (*P *< 0.006 and *P *< 0.02, respectively). No significant difference was observed between the other thyroid hormones and TSH (*P *> 0.05). It was also noted that, although troponin-I and CK-MB values were high, LVEF was low in the CPR subgroup (*P *< 0.03, *P *< 0.02 and *P *< 0.05, respectively; Table 4). At the 2-month follow-up visit, T_3 _and free T_3 _levels were similar between the CPR-alone and cardioversion-alone subgroups (T_3_, 1.12 ± 0.18 versus 1.17 ± 0.28 ng/ml; free T_3_, 0.28 ± 0.94 versus 0.29 ± 0.9 ng/ml; *P *> 0.05).

When the duration of cardiac arrest was considered, it was observed that T_3 _(0.6 ± 0.15 versus 0.93 ± 0.31 ng/ml) and free T_3 _(0.11 ± 0.03 versus 0.24 ± 0.11 ng/ml) levels were lower in patients with interventions of more than 10 min than in those with interventions of less than 5 min (*P *< 0.001). Similarly, TSH (8.9 ± 6.1 versus 13.9 ± 5.8 μIU/ml; *P *< 0.05) and T_4 _(6 ± 1.2 versus 8.5 ± 2.4 μg/dl; *P *< 0.005) levels were lower in those who had interventions of more than 10 min.

Although the day 1 values for thyroid hormones and TSH were lower in the AMI group than in the control group, the difference was not significant (*P *> 0.05). However, day 3 levels of T_3 _and free T_3 _were significantly lower in the AMI group than in the control group (*P *< 0.01). In contrast, serum levels of T_4_, free T_4 _and TSH did not differ significantly between these groups (*P *> 0.05). Thyroid hormones and TSH were lower on day 3 than on day 1 for the AMI group. However, only free T_3 _levels were significantly lower on day 3 when the day 1 and day 3 values were compared (*P *< 0.05; Table 5). T_3 _and free T_3 _values of the patients who died within the first 2 months in the cardiac arrest group were markedly lower than those in survivors (*P *= 0.02 and *P *= 0.03, respectively). T_4_, free T_4 _and TSH levels were low in patients who died, but this finding was not statistically significant (*P *> 0,05). It was also observed that the troponin and CK-MB values in those who died were higher than in survivors, but the LVEF value was lower (*P *< 0.001; Table 6).

When the 2-month TFTs for the cardiac arrest and AMI groups were compared with those in the control group, it was found that the level of free T_3 _(control 0.32 ± 0.02 ng/ml, cardiac arrest 0.29 ± 0.09 ng/ml, AMI 0.29 ± 0.05 ng/ml; *P *> 0.05) and TSH (control 1.2 ± 0.5 μIU/ml, cardiac arrest 1.25 ± 0.48 μIU/ml, AMI 1.27 ± 0.82 μIU/ml; *P *> 0.05) were similar in all three groups. In contrast, the level of T_3 _was lower both in cardiac arrest and AMI groups than in the control group. However, T_3 _in all groups was within the normal reference range (control 1.32 ± 0.28 ng/ml, cardiac arrest 1.15 ± 0.24 ng/ml, AMI 1.18 ± 0.23 ng/ml; *P *< 0.05).

The 2-month follow-up visit revealed that depressed T_3 _and free T_3 _levels in two patients, who were in vegetative state, had persisted. Furthermore, one of those patients was observed to have lower T_4 _and free T_4 _levels, but the TSH level did not change significantly.

## Discussion

To the best of our knowledge, no other published study has demonstrated major alterations in standard thyroid homeostasis during the acute stage of cardiac arrest, which then normalized by the second month in patients who survived cardiac arrest induced by ACS. In severe illnesses of nonthyroid origin [10,11], including cardiac diseases [12], downregulation of the thyroid hormone system can occur. This condition, which has been called the ESS or the 'low T_3 _syndrome', is characterized by a change in thyroid homeostasis. This condition occurs as a result of impairment in the normal feedback response due to low T_3 _levels and disruption in conversion of precursor hormone T_4 _to T_3_. The significantly lower T_3 _and free T_3 _levels in the cardiac arrest group than in the uncomplicated AMI group noted here reflects the critical changes in thyroid homeostasis that occur in cardiac arrest

The hypothalamohypophysial–thyroid axis must function properly to ensure normal thyroid homeostasis. We had postulated that this axis would be disrupted in patients with cardiac arrest caused by impairment in the circulation to the hypophysis and hypothalamus, which would lead to significant TFT abnormalities. In fact, the study revealed that while T_3 _and free T_3 _levels were significantly lower in the cardiac arrest group, TSH was lower as well, albeit it not significantly so. In general, TSH rises in response to lower T_3 _levels. However, in cardiac arrest patients this was not found to be the case, which confirmed the occurrence of ESS in these cardiac arrest patients. Although hormonal changes were more prominent in the cardiac arrest group than in the AMI group, the changes in the two groups paralleled each other. The fact that the changes in thyroid function were observed to return to normal at the 2-month follow-up visit was another indication of the presence of ESS in cardiac arrest. It is known that thyroid functions normalize in ESS patients following improvement in the pathology causing ESS [9,13]. However, it must be noted that some of the patients, who had undergone CPR for a lengthy period, died within the first 2 months. This might have contributed to the difference in results. Normally, secondary hypothyroidism is expected in severe ischaemia of the hypophysis [30]. However, a possible explanation for our findings, characterized by ESS, are as follows: even during critical hypotension, brain perfusion continues via autoregulation of cerebral blood flow, and this prevents more severe complications in intracerebral organs.

Various vasoactive substances have been described that contribute to the physiological regulation of cerebral perfusion, either by vasoconstriction or by vasodilatation [31]. In particular, during severe hypotension, nitric oxide mediated autoregulation has been suggested to play an important role in maintaining brainstem perfusion, which is needed to preserve the integrity of vital brainstem functions [32]. Although cerebral blood flow is inadequate, brain perfusion continues during effective CPR. Therefore, ESS, rather than secondary hypothyroidism, may occur during shorter cardiac arrest events. However, in patients with longer durations of resuscitation, a clinical picture resembling that of secondary hypothyroidism may be observed [30]. In our study, TFT findings in the patients with longer arrest intervals were more impaired.

There are some differences between our study and some others investigating thyroid function in cardiac arrest patients. Regardless of resuscitation success, Longstreth and coworkers [24] observed low T_3 _and T_4 _levels and high TSH levels in patients with out-of-hospital cardiac arrest. They stated that these alterations in thyroid hormones may play a role in cardiac arrest aetiology and prognosis. Wortsman and coworkers [25] reported significantly depressed T_3 _and T_4 _levels. Likewise, T_3_, free T_3_, T_4 _and free T_4 _levels were reported to have decreased in animal studies [26,27]. However, when all patients are considered, our study demonstrated significantly lower T_3 _and free T_3 _in the cardiac arrest group, but no significant changes in T_4_, free T_4 _and TSH levels. However, the lower T_4 _levels observed in subgroup analyses in patients with longer resuscitation periods is consistent with those studies. Meanwhile, one of our patients in a vegetative state had lower T_4 _and free T_4 _values, as well as lower T_3 _and free T_3_. Therefore, we may conclude that T_4 _levels decrease, along with the decrease in the active hormone T_3 _in association with impairment in the hypothalamus–hypophysis–thyroid axis, particularly in patients with longer resuscitation periods. In 42% of pituitary apoplexy cases of various causes (haemorrhage, radiation, intracranial hypertension, etc.), secondary hypothyroidism developed [30]. The length of time in resuscitation may be one of the reasons for the different findings observed in the present study. Furthermore, our study group was homogenous because it comprised patients with cardiac arrest induced by ACS. Lack of a homogenous population in previous studies might have led to inconsistencies between the studies.

ESS may be observed in different forms. A milder form of ESS may be observed with only a decrease in T_3_, as in uncomplicated AMI, and a T_4 _decrease accompanying decreased T_3 _levels may also be observed, as was the case in cardiac arrest patients with longer CPR sessions in the present study. It is known that this condition is associated with increased mortality. Rarely, an increase in T_4 _may also be observed [13,14]. Moreover, it is known that an increase occurs normally at the level of reverse T_3_ in ESS, although we have not measured it. The cause of the decreased T_3 _in ESS has not been established. It has been attributed to various parameters, including test artifacts, inhibitors of T_4 _and T_3 _binding to proteins, decreased 5'-deiodinase activity and circulating cytokines. It is known that inflammation plays a critical role in the pathophysiology of the ESS that occurs in AMI. In particular, interleukin-6 plays a major role in the development of this syndrome. It inhibits conversion of T_4 _to T_3 _by inhibiting mRNA expression or by blocking 5'-deiodinase activity. This inhibition occurs both in the pituitary–thyroid axis and in peripheral transformation of the thyroid hormone [15,16]. Furthermore, Fliers and coworkers [17] reported a strong correlation between thyroid-releasing hormone gene expression and serum T_3 _and TSH concentrations in patients with various degrees of ESS. It is not known whether different mechanisms are involved in the changes that occur in TFTs during cardiac arrest.

The changes observed in thyroid function in the AMI group were characterized by a milder form of ESS and were consistent with previous studies [12,15,19]. However, Pavlou and coworkers [18] reported depressed T_3_, T_4_, free T_3_, free T_4 _and TSH serum levels in complicated AMI. Moreover, those authors maintained that ESS occurred both in AMI and in unstable angina pectoris, and they had suggested an association between the drop in T_3 _and cardiac damage.

Although downregulation of thyroid hormones occurring both in cardiac arrest and AMI groups may be regarded as an adaptive measure to decrease the cardiac workload and conserve energy during acute ischaemia, this effect continues in an unstable manner that then becomes maladaptive [19]. It is known that thyroid hormones have beneficial effects on cardiac contractility, output, systemic vascular resistance and diastolic functions [20-22]. Changes in thyroid hormones that occur because of cardiac arrest or AMI lead to critical haemodynamic alterations in the cardiovascular system by increasing the vascular resistance and decreasing cardiac output [20-23]. In particular, the decrease in active hormone T_3 _leads to further impairment in cardiac functions. Iervasi and coworkers [23] reported low serum T_3 _levels as an independent predictor of mortality in patients with cardiovascular disease. Alterations in TFTs are more marked in seriously ill patients [24-27]. In the present study the TFT findings in those who died within the first 2 months deteriorated more than did those in survivors.

Thyroid hormone replacement therapy has been considered as a result of favourable changes that occur in cardiac functions and cardiac gene expression following T_3 _administration in patients with ESS. Whitesall and coworkers [33] reported that T_3 _replacement did not have positive effects on cardiac function in dogs, but several previously conducted studies demonstrated that T_3 _replacement improved left ventricular function and normalized T_3_-responsive gene expression [26,27,34-39]. Similarly, increased LVEF values as a result of T_3_ administration following AMI was reported in animal studies [26,27,35]. Moreover, it was observed in open heart surgery that T_3 _improved haemodynamic parameters [36,39]. Left ventricular function is among the leading indicators of prognosis following AMI [40]. Furthermore, cardiogenic shock occurring in cardiac arrest and AMI patients is a critical predictor of mortality [41]. Cardiac output decreases significantly because of shock, and if thyroid dysfunction accompanies this then further functional impairments can be expected. Taniguchi and coworkers [8] established in donors with brain death that administration of T_3 _along with cortisol increased blood pressure and had a favourable, stabilizing effect on cardiac function. These studies show T_3 _to be a potential therapeutic approach to improving left ventricular function in ESS [26,27,34-41]. Nevertheless, large-scale studies of T_3_ therapy are required in the setting of haemodynamic instability following cardiac arrest and AMI. One of the limitations of the present study was the fact that some of our patients were administered drugs that could alter TFTs. However, a previous study in AMI patients [18] reported that β-blockers and thrombolytic therapy did not alter thyroid function. Only four patients were administered dopamine. Furthermore, we were unable to document ischaemia of the hypophysis or hypothalamus. Therefore, more studies are required to establish the extent of ischaemia of the hypophysis and hypothalamus in patients undergoing CPR and to investigate its impact on thyroid hormones. Another limitation of the study is that we did not measure the level of reverse T_3 _– an inactive metabolite with prognostic value.

## Conclusion

TFTs are significantly altered in cardiac arrest induced by ACS. The changes in TFTs are even more pronounced in patients with longer periods of resuscitation. The changes in the surviving patients are characterized by ESS and improve by 2 months in patients who have not progressed to a vegetative state. Large-scale studies in cardiac arrest are required to demonstrate the course of TFTs, including measurement at 24 hours and of reverse T_3 _levels.

## Key messages

• 	Thyroid function tests are significantly altered in cardiac arrest induced by ACS in acute stage.

• 	The changes in TFTs are even more pronounced in patients with longer periods of resuscitation.

• 	The changes in the surviving patients are characterized by euthyroid sick syndrome.

• 	These changes in acute stage improve dramatically by the end of the second month.

## Abbreviations

ACS = acute coronary syndrome; AMI = acute myocardial infarction; CK-MB = creatine kinase MB isoenzyme; CPR = cardiopulmonary resuscitation; ESS = euthyroid sick syndrome; ICU = intensive care unit; LVEF = left ventricular ejection fraction; T_3 _= tri-iodothyronine; T_4 _= thyroxine; TFT = thyroid function test; TSH = thyroid-stimulating hormone.

## Competing interests

The author(s) declare that they have no competing interests.

## Authors' contributions

KI created and designed the study, drafted the manuscript, performed the statistical analysis and interpretation of data, and revised the manuscript. GO was involved in the collection, statistical analysis and interpretation of the data. ZA and TT conducted patient monitoring and data collection. NT contributed to the design and the coordination of the study as well as interpretation of the data. All authors read and approved the final manuscript.

## Figures and Tables

**Table 1:** Patient characteristics

	CA (a)	AMI (b)	Control (c)	*P*
Number	50	31	40	-
Age (years)	59 ± 8	57 ± 9	58 ± 6	NS
Sex (male/female)	35/15	25/6	28/7	-
LVEF (%)	44.1 ± 8.2**	48.2 ± 8.6	65.9 ± 3.7*	*c versus a, b **a versus b
Peak troponin I (μg/ml)	29.9 ± 26.1*	6.7 ± 1.6*	< 0.01	*a versus b, c b versus c
Peak CK-MB (IU/l)	228.7 ± 147.4**	170.5 ± 61.2	14.6 ± 4.1*	* c versus a, b **a versus b

*P < 0.0001, **P < 0.05. AMI, acute myocardial infarction; CA, cardiac arrest; CK-MB, creatine phosphokinase MB isoenzyme; LVEF, left ventricular ejection fraction; NS, not significant.

**Table 2:** Thyroid hormones and thyroid-stimulating hormone levels in the controls and cardiac arrest (day 3) and acute myocardial infarction (day 3) patients

	CA day 3 (a)	AMI day 3 (b)	Control (c)	*P*
Number	50	31	40	-
T_3 _(ng/ml)	0.83 ± 0.3*	1.12 ± 0.24	1.32 ± 0.28**	*a versus b, c **b versus c
Free T_3 _(ng/ml)	0.19 ± 0.11*	0.27 ± 0.06	0.32 ± 0.06	*a versus b, c
T_4 _(μg/dl)	7.6 ± 2.3	8.3 ± 1.6	8.4 ± 1.8	NS
Free T_4 _(ng/dl)	1.21 ± 0.5	1.35 ± 0.2	1.28 ± 0.2	NS
TSH (μIU/ml)	1.22 ± 0.6	1.31 ± 0.8	1.2 ± 0.5	NS

*P < 0.0001, **P < 0.01. AMI, acute myocardial infarction; CA, cardiac arrest; NS, not significant; T_3_, tri-iodothyronine; T_4_, thyroxine; TSH, thyroid-stimulating hormone.

**Table 3:** Thyroid hormone and thyroid-stimulating hormone values for cardiac arrest and acute myocardial infarction groups at day 3 and month 2

	CA day 3 (a)	CA month 2 (b)	AMI day 3 (c)	AMI month 2 (d)	*P*
Number	50	28	31	30	
T_3 _(ng/ml)	0.83 ± 0.3*	1.15 ± 0.24	1.12 ± 0.24	1.18 ± 0.23	*a versus b
Free T_3 _(ng/ml)	0.19 ± 0.11*	0.29 ± 0.09	0.27 ± 0.06	0.29 ± 0.05	*a versus b
T_4 _(μg/dl)	7.62 ± 2.34	8.24 ± 2.4	8.27 ± 1.52	8.47 ± 1.5	NS
Free T_4 _(ng/dl)	1.23 ± 0.46	1.25 ± 0.27	1.35 ± 0.2	1.37 ± 1.65	NS
TSH (μIU/ml)	1.22 ± 0.58	1.25 ± 0.48	1.31 ± .0.83	1.27 ± 0.82	NS

*P < 0.0001. AMI, acute myocardial infarction; CA, cardiac arrest; NS, not significant; T_3_, tri-iodothyronine; T_4_, thyroxine; TSH, thyroid-stimulating hormone.

**Table 4:** Day 3 values for cardiac arrest subjected to cariopulmonary resuscitation alone and cardioversion alone

	CPR	CV	*P*
Number	28	22	-
T_3 _(ng/ml)	0.73 ± 0.24	0.94 ± 0.29	<0.006
Free T_3 _(ng/ml)	0.16 ± 0.09	0.23 ± 0.05	<0.02
T_4 _(μg/dl)	7.23 ± 2.34	8.1 ± 2.28	NS
Free T_4 _(ng/dl)	1.15 ± 0.4	1.29 ± 0.5	NS
TSH (μIU/ml)	1.09 ± 0.5	1.38 ± 0.6	NS
Troponin I (μg/ml)	37.3 ± 28.9	20.5 ± 18.7	<0.03
CK-MB (IU/l)	271.8 ± 161.3	173.8 ± 107.7	<0.02
LVEF (%)	42.1 ± 7.9	46.8 ± 7.8	<0.05

CPR, cardiopulmonary resuscitation; CK-MB, creatine kinase MB isoenzyme; CV, cardioversion; LVEF, left ventricular ejection fraction; NS, not significant; T_3_, tri-iodothyronine; T_4_, thyroxine; TSH, thyroid-stimulating hormone.

**Table 5:** Thyroid hormone and thyroid-stimulating hormone values for the control group and acute myocardial infarction group on days 1 and 3

	AMI day 1 (*n *= 31; a)	AMI day 3 (*n *= 31; b)	Control (*n *= 40; c)	*P*
T_3 _(ng/ml)	1.23 ± 0.25	1.12 ± 0.24*	1.32 ± 0.28	*b versus c
Free T_3 _(ng/ml)	0.31 ± 0.06^†^	0.27 ± 0.06^‡^	0.32 ± 0.06	^†^a versus b ^‡^b versus c
T_4 _(μg/dl)	8.4 ± 1.7	8.3 ± 1.6	8.4 ± 1.8	NS
Free T_4 _(ng/dl)	1.38 ± 0.2	1.35 ± 0.2	1.28 ± 0.2	NS
TSH (μIU/ml)	1.35 ± 0.9	1.31 ± 0.8	1.2 ± 0.5	NS

*P = 0.002, ^†^*P *< 0.05, ^‡^*P *= 0.003. AMI, acute myocardial infarction; CA, cardiac arrest; NS, not significant; T_3_, tri-iodothyronine; T_4_, thyroxine; TSH, thyroid-stimulating hormone.

**Table 6:** LVEF, TFTs, troponin and CK-MB levels in the cardiac arrest group, subdivided into those who died and those who survived the first 2 months

	CA survivors	CA died	*P*
Number	28	22	-
T_3 _(ng/ml)	0.9 ± 0.31	0.72 ± 0.18	0.02
Free T_3 _(ng/ml)	0.22 ± 0.12	0.15 ± 0.08	0.03
T_4 _(μg/dl)	8.1 ± 2.5	7.02 ± 2	NS
Free T_4 _(ng/dl)	1.27 ± 0.5	1.14 ± 0.5	NS
TSH (μIU/ml)	1.35 ± 0.5	1.05 ± 0.6	NS
Troponin I (μg/ml)	15.2 ± 9.8	48.6 ± 28.5	<0.0001
CK-MB (IU/l)	148.7 ± 86	330.5 ± 147.7	<0.0001
LVEF (%)	48.4 ± 7.5	38.7 ± 5.3	<0.0001

AMI, acute myocardial infarction; CA, cardiac arrest; CK-MB, creatine kinase MB isoenzyme; LVEF, left ventricular ejection fraction; NS, not significant; T_3_, tri-iodothyronine; T_4_, thyroxine; TSH, thyroid-stimulating hormone.
